# What Concept of Manual Therapy Is More Effective to Improve Health Status in Women with Fibromyalgia Syndrome? A Study Protocol with Preliminary Results

**DOI:** 10.3390/ijerph20021061

**Published:** 2023-01-06

**Authors:** Carine Romane Audoux, Cecilia Estrada-Barranco, Oliver Martínez-Pozas, Rodrigo Gozalo-Pascual, Juan Montaño-Ocaña, David García-Jiménez, Gonzalo Vicente de Frutos, Elena Cabezas-Yagüe, Eleuterio A. Sánchez Romero

**Affiliations:** 1 Musculoskeletal Pain and Motor Control Research Group, Faculty of Sport Sciences, Universidad Europea de Madrid, 28670 Madrid, Spain; 2 Department of Physiotherapy, Faculty of Sport Sciences, Universidad Europea de Madrid, Villaviciosa de Odón, 28670 Madrid, Spain; 3 Escuela Internacional de Doctorado, Department of Physical Therapy, Occupational Therapy, Rehabilitation and Physical Medicine, Universidad Rey Juan Carlos, 28933 Alcorcón, Spain; 4 Facultad de Enfermería, Fisioterapia y Podología, Universidad Complutense de Madrid, 28040 Madrid, Spain; 5 Musculoskeletal Pain and Motor Control Research Group, Faculty of Health Sciences, Universidad Europea de Canarias, 38300 La Orotava, Canary Islands, Spain; 6 Department of Physiotherapy, Faculty of Health Sciences, Universidad Europea de Canarias, 38300 La Orotava, Canary Islands, Spain; 7 OnelifeCenter, Multidisciplinary Center for the Prevention and Treatment of Pain, 28924 Alcorcón, Spain; 8 Department of Physical Therapy, Faculty of Medicine, CEU-San Pablo University, 28668 Madrid, Spain

**Keywords:** fibromyalgia, pain, myofascial, psychological factors, quality of life, anxiety, depression

## Abstract

Background: Fibromyalgia (FM) is defined as a chronic syndrome characterized by diffuse musculoskeletal pain, associated with characteristic signs and symptoms such as fatigue and/or sleep and mood disorders, and whose etiology, pathogenesis and prognosis may or may not be known. There is growing evidence of manual therapy as a treatment for pain in the short and medium term, also in patients affected by FM. However, the heterogeneity of the manual therapy treatments administered are a very common clinical practice, as they are based more on the judgment or tendency of the physiotherapist, rather than on clear scientific evidence. Therefore, the aim of the present study protocol will be to determine which manual therapy approach is more effective in addressing health status by improving symptoms (sensory, cognitive, emotional and social) in patients with FM. Methods: a randomized controlled clinical trial with a 3-month follow-up will be carried out with 52 female patients affected by rheumatologist-diagnosed FM will be recruited and evaluated at the Asociación de Fibromialgia y Síndrome de Fatiga Crónica (AFINSYFACRO) in Móstoles, Madrid, Spain. For more details on the protocol, a pilot study was carried out using a non-probability method of judgmental or purposive sampling. Thirteen patients were also evaluated, treated and reevaluated; eight patients were assigned to the myofascial techniques approach (MTA) group and five to the Maitland’s mobilization approach (MMA) group. Results: the preliminary results presented here are intended to show how the planned randomized controlled clinical trial will develop. Patients who received MTA had significantly improved pain and health status outcomes after treatment and at 1-month follow-up, with no significant change in those who received MMA. Conclusions: the exact details of the study protocol on which the manual therapy approach is more effective in addressing health status by improving symptoms (sensory, cognitive, emotional, and social) in patients with FM are presented. Preliminary results show that manual therapy is effective in improving pain and health status in patients with fibromyalgia at short and medium term, with significant results in those who received MTA.

## 1. Introduction

Fibromyalgia (FM), recognized as a clinical entity by the World Health Organization in the tenth revision of the International Classification of Diseases (1991) [1], is defined as a chronic syndrome characterized by diffuse musculoskeletal pain, associated with characteristic signs and symptoms such as fatigue and/or sleep and mood disorders. The etiology, pathogenesis and prognosis of FM are not fully understood [2,3,4].

The worldwide prevalence of this syndrome is 2.1%, being higher in women than in men with a ratio of 4:16. In the USA there are 26,400,000 people affected by FM [4]. Data close to the global figures are observed in Spain, where it affects 2.4% of the population over 20 years of age, mostly women [2]. 

It involves high direct costs (medical visits, complementary tests, pharmacological and non-pharmacological treatments) and indirect costs (reduction in working hours, sick leave and permanent disability). The cost per patient/year, in studies carried out in the USA and Spain, is between 7813 EUR and 9982 EUR, which implies an annual cost of more than 12,993 million euros [5]. 

This pathology does not have a firm and recognized organic etiology, so its pathogenesis is not yet fully defined. However, some authors postulate that various factors could be involved, among them: dysfunctions of the nervous, immune and endocrine system, environmental stressors and psychiatric or psychological issues [6].

Fibromyalgia is considered part of the group of central sensitization syndromes [7], and is defined as a hyperreactivity or amplified response to a stimulus by the nociceptive neurons in the CNS, and its etiology is multifactorial [8,9]. Although the pathophysiology of this process is still unclear, the windup phenomenon (increased neuronal excitability at the medullary level) and the alteration of descending inhibitory systems (modulators of the medullary response to nociceptive stimuli) [9] are considered.

Patients with FM presents a wide variety of symptoms and signs; among the most frequent are diffuse musculoskeletal pain, primary and secondary hyperalgesia, allodynia, stiffness, fatigue, asthenia, anxiety, depression, and sleep and mood disorders. In addition, it usually manifests together with concomitant pathology such as irritable bowel disease, headaches, neurological or rheumatic disorders or temporomandibular joint disorders [6,8,10,11].

The use of conventional analgesics is not usually effective in pharmacological treatment and so-called pain-modulating drugs, including antidepressants and antiepileptics, are prescribed; however, the proportion of patients who achieve an improvement in symptoms is very small [12]. 

There is high evidence of treatment combining exercise, aquatic exercise, other active therapies and multimodal therapies, on improving pain intensity, disability and physical function in the short term. In addition, manual therapy, needle therapies and patient education also provide short-term benefits [13].

In recent years, evidence is growing that manual therapy is a medium-term treatment for pain, also in patients affected by fibromyalgia [14,15,16,17]. However, the heterogeneity of the manual therapy treatments administered are a very common clinical practice, as they are based more on the judgment or tendency of the physiotherapist, rather than on clear scientific evidence.

Although the mechanisms of manual therapy have not been fully elucidated, its effect is explained by a mechanical stimulus that initiates a cascade of peripheral and central neurophysiological effects that are related to a decrease in the concentration of proinflammatory mediators, in addition to a decrease in mechanical hyperalgesia, and a stimulation of the supraspinal inhibitory pathways to produce a hypoalgesic response [14,15,16,17,18].

A recent published systematic review and meta-analysis [19] concluded that the myofascial techniques approach (MTA) improves pain, sleep, and quality of life compared to sham in patients with FM.

Accessory joint mobilizations (also known as Maitland´s Mobilizations) reduce pain, improve range of motion, and have effects that reflect an improvement in the autonomic profile through increased vagal activity, as well as an improvement in psychological factors associated with FM. [15,16].

Therefore, the aim of the present study is to determine which of the most commonly used manual therapy approaches in clinical practice is more effective in addressing health status by improving symptoms (sensory, cognitive, emotional and social) in patients with fibromyalgia.

## 2. Materials and Methods

### 2.1. Study Design

**Figure 1:**
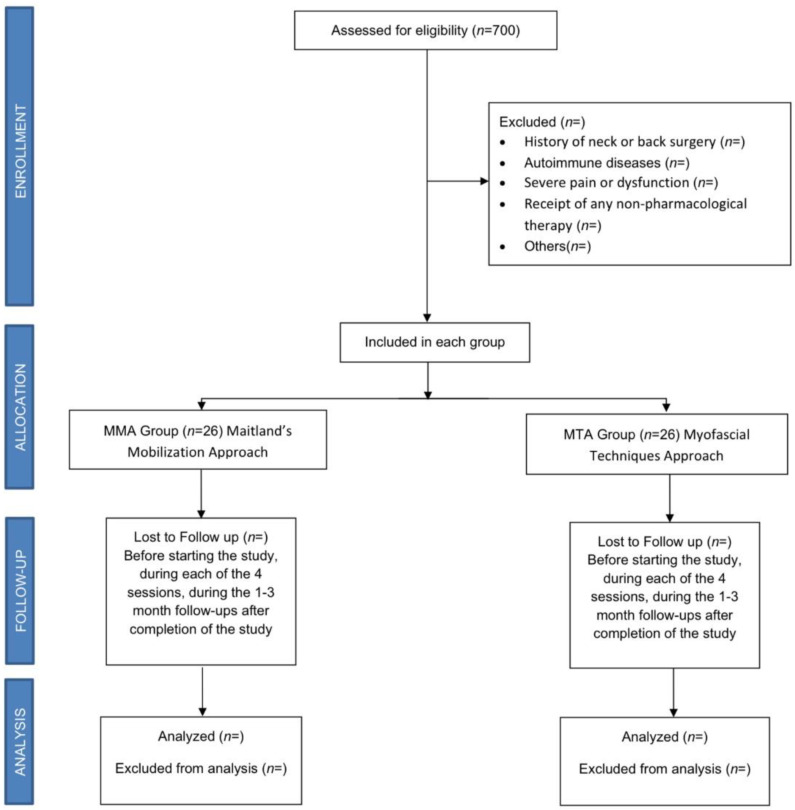
Consort flow diagram.

A randomized controlled clinical trial (RCCT) with a 3-month follow-up will be carried out between January 2023 and July 2023, with 52 female patients affected by rheumatologist-diagnosed FM who will be recruited and evaluated at the Asociación de Fibromialgia y Síndrome de Fatiga Crónica (AFINSYFACRO) in Móstoles, Madrid, Spain. The study protocol was approved by the Ethical Committee of the Rey Juan Carlos University, Madrid, Spain (reference number 2609202220822). In addition, the study was registered with ClinicalTrials.gov (NCT05559021), and will be conducted in accordance with CONSORT Statement 2010: updated guidelines for reporting parallel group randomized trials (CONSORT 2010 flowchart, Figure 1). According to the Declaration of Helsinki, all patients will sign an informed consent form before inclusion and must agree that their clinical information will be published anonymously. 

For more details on the protocol, a pilot study was carried out between January 2022 and July 2022 by recruiting thirteen patients using a non-probability method of judgmental or purposive sampling. The study protocol was approved by the Ethical Committee of the Rey Juan Carlos University, Madrid, Spain (reference number 2609202220822). All patients signed an informed consent form before inclusion.

### 2.2. Sample

Fifty-two consecutive patients younger than 65 years who meet the American College of Rheumatology (ACR) clinical criteria for FM [20] will be included in the study. Patients will be recruited by the center’s staff, who will be blinded to the severity of Fibromyalgia assessment performed. The center’s staff will be in charge of promoting the study through their direct contact, both in group activities and through individual talks. Individuals who will be included in the study will have a minimum one-year history of symptoms. To this medical diagnosis will be added a clinical evaluation based on the 2010 American College of Rheumatology (ACR) criteria [21], performed by the physical therapists and investigators participating in the study (J.M.O. and G.V.F.): given that 2 of the measurement variables that will be used in the study (widespread pain index, WPI; and symptom severity index, SS-Score) coincide with the protocol, when determining these criteria, the diagnosis of fibromyalgia will be corroborated when WPI ≥ 7 and SS ≥ 5 or WPI 3–6 and SS ≥ 9 [22]. The exclusion criteria will be: the receipt of any non-pharmacological therapy, presence of cardiac, renal or hepatic failure, severe physical disability, comorbidities (e.g., interstitial cystitis, inflammatory disease), infection, fever, hypotension, respiratory disorders limiting treatment, herpes, lupus, multiple sclerosis, rheumatoid arthritis, polio, epilepsy, rheumatic fever, cancer, history of neck or back surgery, skin disorders, psychiatric disease, non-compliance with the prescribed drug therapy, and pregnant women or with the possibility that they could be pregnant due to the incompatibility of the techniques [14,15,16,17,23].

In addition, out of twenty-three patients, thirteen finally met all the requirements and criteria and were recruited to present preliminary results by conducting a pilot study in order to provide a clearer description of the protocol.

#### Sample Size

The latest version of the free GRANMO program version 7.12 was used. The sample size of 52 randomly distributed patients will provide at least 80% statistical power to detect a difference of 14 units between the two groups in the FIQ inventory. This calculation assumes a bilateral significance level of 5% and a standard deviation of 17. In addition, a loss rate of 10% was calculated until the end of the study.

### 2.3. Intervention

There will be two intervention groups: myofascial techniques approach (MTA) group and Maitland’s mobilization approach (MMA) group. Both interventions were conceived and were (pilot study) and will be (RCCT) developed by clinicians and professors of Manual Therapy at the European University of Madrid (Spain), the Complutense University of Madrid (Spain), and CEU-San Pablo University of Madrid (Spain), with more than 15–20 years of clinical experience (C.E.B.; O.M.P.; R.G.P.; D.G.J.; E.C.Y.; and E.A.S.R.). The research team includes two doctors in physiotherapy and four doctoral students, as well as a doctor in psychology. Only C.A.R. had 2 years of clinical experience and a Master of Science degree.

#### 2.3.1. MMA Group

After a literature review of manual therapy treatments for fibromyalgia patients [16,24,25] and through a consensus of experts with 15–20 years of clinical experience, there was a decision to perform the following passive mobilizations approach for 1 session per week of 30 min each, for a total of 4 weeks.

**Figure 2:**
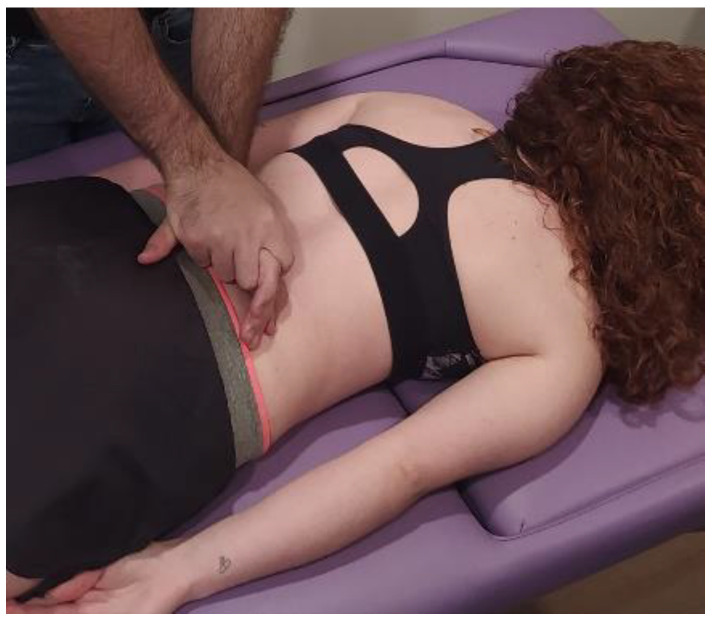
Lumbar mobilization technique: The therapist will perform postero-anterior joint mobilization using Maitland’s technique, applying pressure on the spinous process of the lumbar target vertebra.

**Figure 3:**
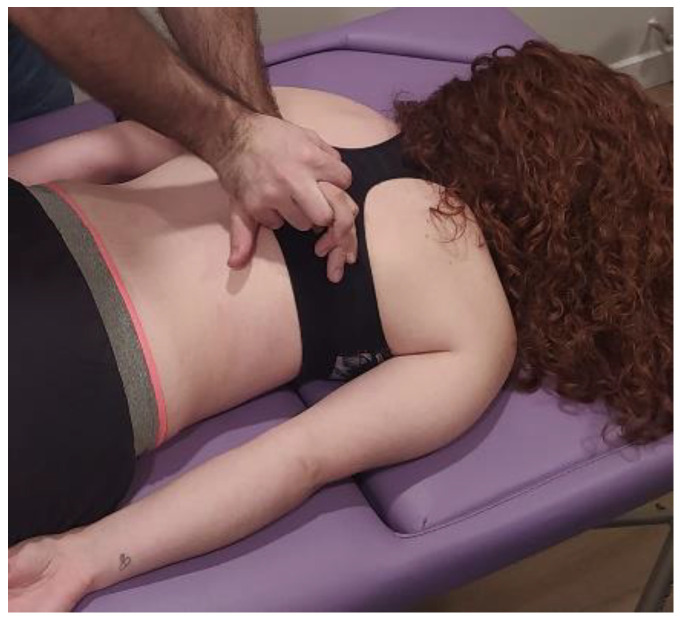
Thoracic spine mobilization technique: The therapist will perform postero-anterior joint mobilization using Maitland’s technique, applying pressure on the spinous process of the thoracic target vertebra.

**Figure 4:**
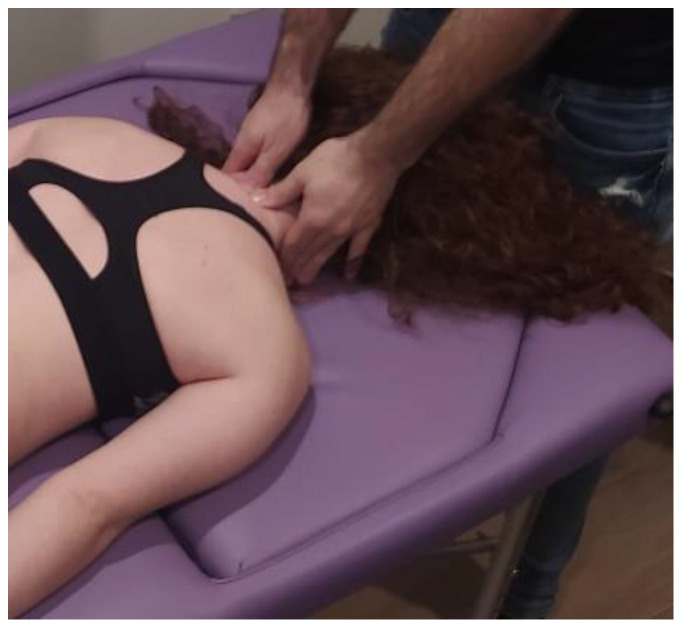
Cervical spine mobilization technique: The therapist will perform postero-anterior joint mobilization using Maitland’s technique, applying pressure on the spinous process of the cervical target vertebra.

The MMA group will be carried out with patient lying in a prone position, with the hands around the body and neck positioned comfortably. The therapist will perform postero-anterior joint mobilization using Maitland’s technique, applying pressure on the spinous process of the target vertebra (the one which most reproduces patient’s symptoms) [24]. Amplitude of mobilization will be done according to patient’s irritability, as other studies recommended [25]. Cycles of joint mobilizations will be applied to lumbar spine (Figure 2), thoracic spine (Figure 3) and cervical spine (Figure 4). 

In addition, it was decided to perform 3 sets of 2 min duration with 1 min of rest in each painful segment of the cervical, thoracic and lumbar spine. Spinal mobilizations have greater global, systemic and pain-modulating reflexogenic effects than peripheral joint mobilizations, so it was decided in expert consensus to select this approach targeting the spine with joint mobilizations [16,23,24,25].

#### 2.3.2. MTA Group

**Figure 5:**
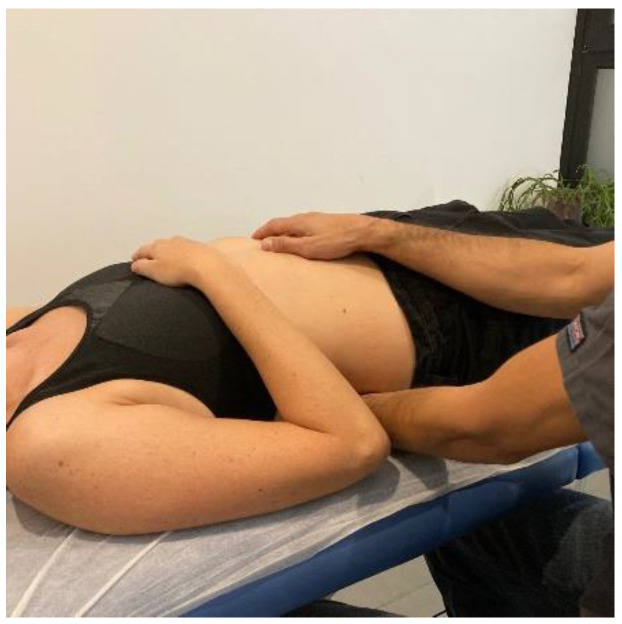
Transverse planes on the thoracolumbar fascia (TCL) and abdomen (myofascial technique 1).

**Figure 6:**
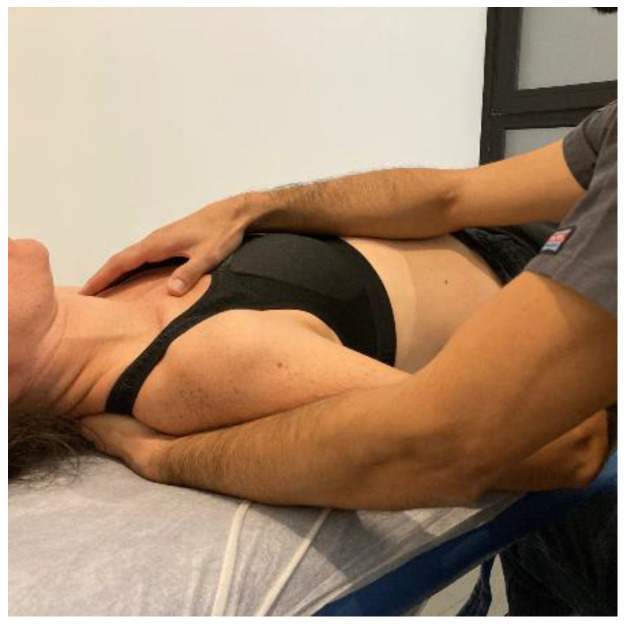
Transverse planes at C7-D3 and sternum (Myofascial technique 2).

**Figure 7:**
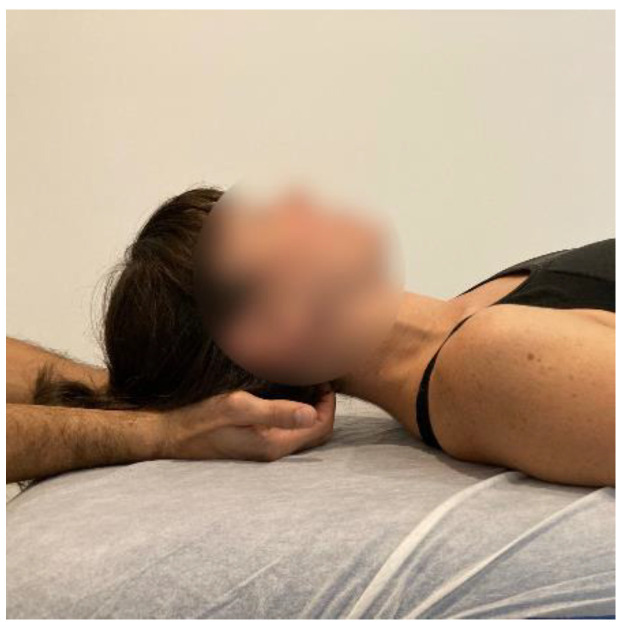
Suboccipital inhibition (myofascial technique 3).

After a literature review of manual therapy treatments for fibromyalgia patients [14,17,19,26] and through a consensus of experts with 15–20 years of clinical experience, it was decided to perform myofascial techniques whose a systemic mechanism of action which best fit the picture of fibromyalgia disease. The MTA approach will consist of the application of the following myofascial techniques under the parameters of low load and long duration to the myofascial complex until the tissue restriction disappears with a frequency of 1 session per week of 30 min each, for a total of 4 weeks:

- Transverse planes on the thoracolumbar fascia (TCL) and abdomen: the physical therapist, seated on one side of the table, places his hands facing each other so that one hand is between the table and the patient’s TCL and the other on the abdomen (leaving the navel between the first commissure) (Figure 5, myofascial technique 1).
- Transverse planes at C7-D3 and sternum: the physical therapist, seated on one side of the table, places his hands facing each other so that one hand is between the table and the patient’s first 4 thoracic vertebrae (C7-T4) and the other on the sternum (thumb and index finger on each of the sternoclavicular joints) (Figure 6, Myofascial technique 2).
- Suboccipital inhibition: in a first phase, the physiotherapist, seated at the head of the table, places his hands under the patient’s head transversely between the occipital and the spinous process of C2, so that the metacarpophalangeal joints are at 90° and the head is suspended with only this contact. In a second phase, the head is lowered in such a way that the occipital bone rests on the tenar eminences and a slight traction is maintained cranially (Figure 7, Myofascial technique 3).

### 2.4. Randomization and Blinding

To ensure adequate blinding, participants will be randomly assigned to either the MMA (Maitland’s mobilization approach) group or the MTA (myofascial techniques approach) group immediately after they sign the informed consent forms. The randomization process will use numbers in sequential opaque envelopes. Patients will be asked not to comment on the treatment to other participants or to the evaluators. Details of adverse events will be recorded in a document which will be given to an administrator and completed separately by subjects in a room before the next session. Graph Pad software will be used to perform randomization, which will include the MMA group and the MTA group [27]. 

Thirteen patients were also evaluated, treated and reevaluated for the pilot study. Using a non-probability method of judgmental or purposive sampling, eight patients were assigned to the MTA group and five to the MMA group.

### 2.5. Outcomes Measurement

The two primary outcomes will be assessed using the two assessment tools used in the 2010 American College of Rheumatology (ACR) FM diagnostic criteria, validated for use in the Spanish population, with a cut-off score of WPI ≥ 7 and SS ≥ 5 or WPI 3–6 and SS ≥ 9 [22]. J.M.O. and G.V.F. will be in charge of the post-treatment evaluation of the subjects, as well as the evaluation at each of the follow-ups, although all the questionnaires included are self-completed. Both members of the research team are clinicians with 10 years of daily experience, as well as professors of manual therapy at the European University of Madrid (Spain).

#### 2.5.1. Widespread Pain

This will be classified as a continuous numerical variable measured by the widespread pain index (WPI). In this index, the patient has to mark with an x the areas in which he/she has presented pain during the last week. The WPI is a valid pain extension scale that was previously described as a regional pain scale and is comprised of a list of 19 painful body areas, where patients indicate whether a specific painful area is painful on a scale ranging from 0 to 19 [28].

#### 2.5.2. Symptom Severity

This variable will be classified as a continuous numerical variable, measured by the symptom severity index (SSI/SS-Score), composed of two subscales (SS-1 and SS-2) that assess the intensity during the last week of 3 of the main symptoms present in FM (fatigue, unrefreshing sleep and cognitive disorders), as well as the presence/absence of other common symptoms, respectively. The total score of the SSS is the sum total and ranges from 0 to 12. The SSS correlated correlates strongly with the WPI (r = 0.733) and with the presence of tender points (r = 0.680) [29].

#### 2.5.3. Impact of FM on Quality of Life

Another measurement variable will be the impact of fibromyalgia on quality of life, through the fibromyalgia impact questionnaire (FIQ), a tool translated into Spanish by means of international translation–retrotranslation recommendations [21], and whose reliability, validity, adaptation and sensitivity to change has been satisfactorily analyzed in Spanish patients with FM [30,31]. This questionnaire consists of 19 items that measure three main categories (a) “function”—ten items that assess the participant’s physical functions that address the participant’s ability to perform each activity. This domain will be assessed on a 4-point Likert scale, from 0 to 3 (0 = always, 1 = frequently, 2 = occasionally, 3 = never). (b) “Global impact”—composed of two items assessing the number of days in the last week when the participants felt good and the number of days they could not work due to FM symptoms.

In addition, the EuroQol 5-dimensions 5-levels (EQ-5D-5L) will be used: a test where mobility, self-care, activities of daily living, pain/discomfort and anxiety/depression are assessed [32]. The patient himself assesses his state of health, in levels of severity by dimensions. The first allows the respondent to define the state of health according to the EQ-5D multi-attribute classification system, composed of 5 dimensions (mobility, self-care, activities of daily living, pain/discomfort and anxiety/depression), and in each of them there are 3 levels of severity (1, 2 or 3).

#### 2.5.4. Assessment of Perceived Pain and Sensitization-Associated

Perceived pain will be measured with the numerical pain rating scale (NPRS), which measures pain intensity [33]. The worst pain intensity at rest were assessed on a 10-point (NPRS, 0: no pain, 10: maximum pain).

The presence of self-reported, sensitization-associated and neuropathic pain symptoms will be assessed with the central sensitization inventory (CSI), which is a 25-item self-report questionnaire [34]. Each item will be evaluated on a 5-point Likert scale for a total of 0 to 100 points. A cut-off value of 40 points suggests an alteration of nociceptive pain processing.

#### 2.5.5. Sleep Quality

Sleep quality will be measured with the Pittsburgh sleep quality index (PSQI) consists of 19 self-administered questions and 5 questions assessed by the patient’s partner or roommate (if available). Only the self-administered questions are included in the score. The PSQI, developed by the Department of Psychiatry at the University of Pittsburgh in 1988, is a questionnaire that assesses both qualitative and quantitative aspects of sleep quality in the month prior to its administration. It shows that subjective sleep quality, duration, efficiency, disturbances and daytime dysfunction have better quality in those with moderate and severe impairment; while in those with average and higher performance, higher sleep latencies are observed [35]. The total score is 21, and scores above 5 indicate significant sleep disturbance.

#### 2.5.6. Physical Activity

The international physical activity questionnaire (IPAQ) will be used to assess physical activity levels [36]. The questionnaire consists of a total of seven questions, related to activities performed in the last seven days prior to the application of the questionnaire. The questions measure the principles of physical activity, such as walking, moderate and vigorous intensity activities, frequency and duration. The data obtained will be converted into MET min/week (i.e., metabolic equivalent task) by calculating the minutes scored per week in each activity category by specific metabolic equivalent in accordance with previous research [37].

#### 2.5.7. Psychological, Cognitive, and Emotional Factors

Finally, psychological, cognitive, and emotional factors will be measured, such as kinesiophobia [38,39], anxiety [40], depression [41], psychological distress [42], and self-efficacy [43,44,45]. For this purpose, we will use the Tampa scale of kinesiophobia (TSK), the state-trait anxiety inventory (STAI), the Beck depression inventory II (BDI-II), and the chronic pain self-efficacy questionnaire (PSEQ), all of them translated and validated in Spanish [45,46]. The TSK is a 17-item self-report measure that will be used to measure fear of movement. A high score indicates an extreme fear of movement, while a low score indicates a negligible fear of movement. The STAI questionnaire is used to assess two independent concepts of anxiety: trait anxiety and state anxiety, each with 20 items: anxiety as a trait indicates a relatively stable anxious propensity that characterizes individuals with a tendency to perceive situations as threatening and it is the one used when studying changes over time and the one used in this study. BDI-II is a useful tool to assess symptoms of depression, both in anxiety disorders and depressive conditions. The questionnaire consists of 21 questions, providing a range of scores between 0 and 63. The questionnaire proposes the following cut-off scores and corresponding degrees of depression: 0–13 indicates minimal depression, 14–19 mild depression, 20–28 moderate depression and 29–63 severe depression. Psychological distress will be measured with the general health questionaire-12 (GHQ-12), which consists of 12 items that survey the individual’s mental health over weeks. It offers four response options and provides scores ranging from 0 to 36, where higher scores are more indicative of mental health problems. The PSEQ is a 10-item questionnaire developed to assess the confidence that people with persistent pain have in performing activities while in pain. The PSEQ is applicable to all presentations of persistent pain. It enquires about the level of self-efficacy in relation to a range of functions, such as housework, socializing and work, as well as coping with pain without medication. 

#### 2.5.8. Patient Satisfaction

To assess patient satisfaction, the global assessment of change (GRoC) will be used [47]. The GRoC is a 15-point scale with a central value of 0 (no change) and values above or to the left ranging from −1 to −7 (much worse), and below or to the right from +1 to +7 (much better). For the study we will consider those patients who score “considerably better” or better (+5 or more) will be those who received successful treatment. It has previously been published that a score of +4 (moderately better) is a suggested cut-off point for dichotomizing improvement versus non-improvement [48].

### 2.6. Statistical Analysis

The distribution of quantitative variables will be analyzed with the Kolmogorov– Smirnov test, to evaluate the normality of the samples to verify if there are significant differences in the two samples at baseline, as well as at different times (*p* > 0.05). The t-test will be used for comparison of characteristics between the different groups at baseline. The chi-square test will be used to assess the independence of the categorical baseline data. The mean, SD and 95% CI will be calculated for all variables. Repeated-measures analyses of variance (ANOVA) with two factors (2 (treatment groups) × 5 (time: baseline, immediately after intervention, and at one month, two months and three months after the study started)) will be performed for the FIQ and NPRS scores. The comparisons for time factor for all variables and for each group will be applied to the group. Time comparisons for all variables and for each group the proportion of subjects who have an improvement than the NPRS MCID (2.0 points) or the FIQ MCID (14%) will be calculated and will be compared between groups using the *p* value [26,49]. The effect size will be calculated for the NPRS and FIQ variables. *p* values associated with F statistics will be adjusted for ANOVA via the Greenhouse–Geisser correction. Statistical significance will be set at *p* value of <0.05 for all calculations. 

For the analysis of the pilot study, the data were analyzed with the SPSS package version 25.0 (SPSS Inc, Chicago, IL, USA). The homogeneity of the sample in both groups was examined at the beginning of the study with respect to the variables studied to detect statistically significant differences using the Mann–Whitney U test. Descriptive statistics, including frequency counts for categorical variables and measures of central tendency and dispersion for continuous variables, were calculated to summarize the data. Paired-samples T-test was used to compare pre, post, and 1–2-month measurements of all variables studied. The Wilcoxon rank test for related samples was used to establish differences between scores before and immediately after the 4 treatment sessions, 1 month after the intervention and 2 months after the intervention in the two groups. For all study data, *p* values less than 0.05 were considered significant.

## 3. Results

The following are the results of the preliminary application of the protocol in thirteen patients who met the inclusion criteria and were recruited for the pilot study. All completed the intervention and the one- and two-month follow-up evaluation, except for one patient who failed to complete the two-month follow-up. A non-probabilistic method of judgmental or purposive sampling was performed, and eight patients were assigned to the MTA group and five to the MMA group. There were no significant differences between groups in terms of demographic characteristics at the time of the baseline screening (*p* > 0.05). Demographic data are summarized in Table 1.

There were no significant differences between groups in terms of clinical characteristics at the time of the baseline screening (*p* > 0.05). Pre-intervention data are summarized in Table 2.

After the intervention, statistically significant differences were found in the MTA group in the variables NPRS (*p* = 0.018), CSI (*p* = 0.012), GHQ12 (*p* = 0.011), FIQ (*p* = 0.036) and CSI (*p* = 0.012). The rest of the variables studied showed no significant differences in the MTA group. None of the variables studied showed statistically significant differences in the MMA group after the intervention (Table 3).

Similarly, test scores before the intervention were compared with the same scores taken one month after the intervention. In the MTA group, the differences found in the GHQ12 (*p* = 0.05) and FIQ (*p* = 0.036) scales were maintained. In the rest of the variables studied in the MTA group and in all the variables collected in the MMA group, no statistical differences were found at one month. No difference between baseline scores and scores obtained at two months was found in either treatment group (Table 3).

## 4. Discussion

The preliminary results presented here are intended to show how the planned randomized controlled clinical trial will develop. 

However, it is worth commenting that the results obtained in the 13 patients show that, although both groups improved some outcomes after treatment, statistically significant differences were found in lower scores obtained in the 8 patients treated with MTA in the assessment of perceived pain and sensitization-associated (NPRS and CSI), in general health status (GHQ12) and in quality of life measured with the FIQ scale after 4 weeks of treatment. Improvements were maintained 1 month after treatment for general health status (GHQ12) and quality of life measured with the FIQ scale in the 8 patients treated with MTA, but not at 2 months. In contrast, no significant changes were found after the intervention or at any follow-up in MMA group.

These preliminary results, mainly those related to pain, fit with those obtained in the systematic review by Ughreja et al. [19] where there was a large effect on pain reduction immediately after myofascial treatment, which fades to a medium long-term effect. However, both the heterogeneity of the treatments in the articles included in Ughreja et al. [19] review, as well as their low sample size, do not allow conclusions to be drawn in this regard, with standardized MTA protocols being necessary for the treatment of pain as well as other associated symptoms in FM patients.

Regarding MMA intervention, preliminary results found no significant differences after treatment, which is related to other studies which compared joint mobilization with sham and also found no significant differences, although there is a lack of studies evaluating effects of orthopedic manual therapy in patients with FM [50]. 

Future studies should include larger sample sizes, longer follow-up and homogeneous treatments, since different doses were used in different manual therapy studies.

This is the reason for the proposed RCCT, which is intended to respond to a need in the field of manual therapy research in patients with FM. Furthermore, as FM is a disease characterized by high levels of central sensitization in most patients [51], and its clinical approach uses the central sensitization inventory which assesses self-reported symptoms associated with pathologies related to central sensitization [34], all treatment assessment will be complemented by patient satisfaction with GRoC [47,48].

## 5. Conclusions

Here are presented the exact details of an RCCT study protocol on which of the most commonly used manual therapy approaches in clinical practice is most effective in addressing health status by improving symptoms (sensory, cognitive, emotional and social) in patients with FM.

In addition, preliminary results of a pilot study of 13 cases are presented, and it is worth mentioning that improvements were obtained in the 8 patients treated with MTA in pre-post perceived pain and sensitization-associated; as well as in general health status and quality of life after 4 weeks of treatment and at one-month follow-up. 

In the MMA group, no differences were found between baseline and post-treatment measurements, as well as in the measurements taken at 1- and 2-months follow-up.

## Figures and Tables

**Table 1:** Characteristics of the sample.

	MTA Group	MMA Group
	Mean (±SD)	Mean (±SD)
*n*	8	5
Age (y)	58.57 (±5.38)	57 (±9.77)
Time with pain (y)	15.14 (±8.13)	24.2 (±16.2)
Time from diagnosis (y)	8.14 (±4.91)	9.8 (±3.03)
Has a job	Yes: 37.7%; No: 50%; N/A: 12.5%	Yes: 80%; No: 20%
Annual Salary	<25.000: 0 >25.000 > 40.000: 37.5% >40.000: 0 W/S: %: 62.5%	<25.000: 80% >25.000 > 40.000: 0 >40.000: 0 W/S: 20%
Lives Alone	Yes: 87.5%; N/A: 12.5	Yes: 20% No: 80%

MTA group: myofascial techniques approach; MMA group: Maitland’s mobilization approach; SD: Standard deviation; N/A: No answer, W/S: Without salary; (y): years.

**Table 2:** Comparison between groups before intervention.

	MTA Group	MMA Group	U-Mann-Whitney
	Mean (±SD)	Mean (±SD)	*p*
NPRS	6.13 (1.24)	6.40 (0.548)	0.696
IPAQ	1.63 (0.74)	2.20 (0.837)	0.212
GHQ12	8.13 (7.75)	19.20 (3.7)	0.658
EuroQol-5D	0.81 (0.83)	0.681 (11.51)	0.079
BDI-II	2.29 (1.254)	3.20 (0.837)	0.2
STAI	32.75 (11.33)	36.8 (7.62)	0.419
PSQI	14.63 (3.7)	12.4 (4.45)	0.302
FIQ	52.28 (17.25)	54.87 (11.27)	0.884
CSI	5563 (10.65)	63.80 (12.05)	0.213
TSK	25.38 (6.65)	19.40 (2.6)	0.056

MTA group: myofascial techniques approach; MMA group: Maitland’s mobilization approach; NPRS: numerical pain rating scale; IPAQ: international physical activity questionnaire; GHQ12: international physical activity questionnaire; BDI-II: Beck depression inventory II; STAI: State–trait anxiety inventory; PSQI: the Pittsburgh sleep quality index; FIQ: fibromyalgia impact questionnaire; CSI: central sensitization inventory; TSK: Tampa scale of kinesiophobia; SD: standard deviation. * Significantly different within-group, *p* < 0.05 (95% confidence interval).

**Table 3:** Comparison between baseline and post-intervention score, at one and two months.

	MTA Group						MMA Group					
	Mean ± SD			*p* Values			Mean ± SD			*p* Values		
	Post	1 m	2 m	Pre-Post	Pre-1 m	Pre-2 m	Post	1 m	2 m	Pre-Post	Pre-1 m	Pre-2 m
NPRS	3.13 ± 2.29	5.63 ± 2.06	5.64 ± 2.75	0.018 *	0.518	0.735	4.6 ± 24	7.40 ± 0.89	6.6 ± 1.14	0.136	0.129	0.655
IPAQ	2.12 ± 0.835	2.13 ± 0.99	1.57 ± 0.535	0.102	0.102	0.564	2.20 ± 0.44	1.6 ± 0.55	1.8 ± 0.48	1	0.18	0.157
GHQ12	8.25 ± 3.96	12.63 ± 5.01	14.8 ± 4.45	0.011 *	0.05*	0.446	18.6 ± 9.18	22.4 ± 6.1	16 ± 8.27	0.5	0.144	0.416
EuroQol-5D	0.78 ± 0.12	0.76 ± 0.09	0.8 ± 0.15	0.553	0.263	1	0.67 ± 0.15	0.67 ± 0.11	0.72 ± 0.13	0.9	0.893	0.686
BDI-II	1.63 ± 0.744	1.75 ± 0.7	1.43 ± 0.78	0.102	0.317	0.180	2.60 ± 1.52	2.4 ± 0.89	2.20 ± 1.3	0.18	0.102	0.197
STAI	27.25 ± 10.05	28.13 ± 8.04	31.29 ± 8.9	0.107	0.207	0.733	34.2 ± 3.56	37.4 ± 8.56	33.8 ± 6.38	0.273	1	0.465
PSQI	13 ± 4.20	16.13 ± 3.52	15.29 ± 2.21	0.446	0.396	0.461	11.8 ± 4.44	13.6 ± 6.02	14.4 ± 5.46	0.581	0.686	0.496
FIQ	44.07 ± 13.14	41.33 ± 8.20	44.78 ± 11.87	0.036 *	0.036 *	0.31	58.41 ± 24.6	51.66 ± 14.66	49.07 ± 9.57	0.893	0.5	0.5
CSI	49.75 ± 11.46	53.5 ± 12.23	54 ± 6.08	0.012 *	0.324	0.865	62.6 ± 11.13	58.2 ± 18.17	60.4 ± 10.33	0.686	0.345	0.138
TSK	24.63 ± 7.46	24 ± 7.54	22.71 ± 6.72	0.778	0.395	0.672	18.2 ± 3.83	24 ± 9.48	20.8 ± 3.96	0.588	0.5	0.496

MTA group: Myofascial techniques approach; MMA group: Maitland’s mobilization approach; NPRS: numerical pain rating scale; IPAQ: international physical activity questionnaire; GHQ12: international physical activity questionnaire; BDI-II: Beck depression inventory II; STAI: state–trait anxiety inventory; PSQI: the Pittsburgh sleep quality index; FIQ: fibromyalgia impact questionnaire; CSI: central sensitization inventory; TSK: Tampa scale of kinesiophobia; SD, standard deviation. * Significantly different within-group, *p* < 0.05 (95% confidence interval).

## Data Availability

The data presented in this study are available on request from the corresponding authors.
